# Factors Influencing the Integration of Traditional Medicine and Mainstream Medicine in Mental Health Services in West Africa: A Systematic Review Using Narrative Synthesis

**DOI:** 10.1007/s10597-024-01263-w

**Published:** 2024-04-15

**Authors:** Batuuroh I. P. Soori, Krishna Regmi, Yannis Pappas

**Affiliations:** Faculty of Health and Social Sciences, Institute for Health Research, University of Bedfordshire, Luton, LU2 8LE UK

**Keywords:** Integration of traditional medicine, Mainstream medicine, Mental health services, West Africa, Rainbow model of integrated care

## Abstract

This study explored the enablers and obstacles to the integration of traditional medicine and mainstream medicine in mental health services in West Africa. This study is a systematic review conducted in accordance with the relevant parts of the Preferred Reporting Items for Systematic reviews and Meta-analyses. Keywords searches were done in databases, and other reference lists were also searched. The Rainbow model of integrated care and a thematic analysis framework were used to account for the factors influencing the integration of traditional medicine and mainstream medicine in mental health services in West Africa. A total of 12 studies met the eligibility criteria after the evaluation of 6413 articles from databases and reference lists. The themes of: policy and implementation; different conceptualisation of mental health/referrals; trust issues, and education and training, were enablers or obstacles of integration depending on how they worked to facilitate or hinder integration. There was an indication of little integration of TM and MM at the macro, meso and micro levels in mental health services in West Africa. Though the study does cover all the West African states evenly, it is recommended that policy-makers and stakeholders interested in integration should ensure integration activities, especially policies, cut across all the levels of the rainbow model of integrated care and are planned and aligned at the macro, meso and micro levels instead of using ad hoc measures, informal initiatives or placing TM services in MM mental health services, which do not amount to integration.

**Supplementary Information:**

The online version contains supplementary material available at 10.1007/s10597-024-01263-w.

## Introduction

Policy-makers have for ages grappled with how to enable healthcare systems to deliver better health services with limited resources (Valentijn et al., 2015). The World Health Organisation (WHO) and the African Union (AU) have promoted the integration of the two health systems of traditional medicine (TM) and mainstream medicine (MM) in African health systems, as another way of enabling affordable and accessible healthcare for the ever-growing African populations (AU, 2001). Traditional medicine can also be referred to as alternative medicine or non-allopathic therapy, which is the totality of knowledge and practices, whether explicable or not, used in diagnosing, preventing or eliminating physical, mental and social diseases. These include the diverse health practices, pathways, knowledge and beliefs incorporating plant, animal, and mineral-based medicinal products (WHO, 2013, 2019, p. 44). On the other hand, MM, is sometimes referred to as conventional medicine or allopathic therapy, is arguably based on the scientific study of diseases, their symptoms and their management using pharmaceutical products, radiation or surgery by healthcare staff (HCS) and service users (SUs) (National Institutes of Health, 2022)**.**

The development and the integration of TM and MM were based on the WHO regional strategies on promoting and enhancing the role of TM in health systems for 2001–2010 and for 2013–2023, and the AU plan of action (PoA) for the first decade (2001–2010) and for the second decade of African TM (2011–2020) (WHO, 2022). These strategies have, therefore, served as important guides for the integration of TM and MM in African health systems. The move to integrate TM and MM in African health systems was predicated on evidence that up to 80% of Africans use TM for their healthcare needs (WHO, 2002; Kwame, 2021). The AU envisaged integration of TM and MM could help address the health deficits in Africa with the adaptation of the WHO’s strategy and AU’s PoA for the Africa region (AU, 2001) and lower the burden of relying on the MM system (Mokgobi, 2013).

The need for this study is precipitated by three issues: 1, a narrow focus on TM and MM integration in the physical health sector (PHS) at the expense of integration in mental health system (MHS) in West Africa (WA); 2, there are disparate discussions on integration which do not adequately address the factors influencing the integration of TM and MM in MHS in WA; and 3, there is an absence in the literature of the use of integration models in the integration of TM and MM in MHS in WA.

Following the WHO and AU strategies on TM, various attempts have been made to develop and integrate TM and MM; however, these gestures by African states are made from a narrow physical health (PH) lens, with priorities usually given to research and integration of TM and MM in the PHS, exemplified by numerous cases of TM use in the PHS, such as using TM to treat physical health afflictions such as sickle-cell disease and hypertension (WHO, 2000; Kasilo et al., 2013; Kasilo et al., 2019), for treating snakebites (Steinhorst et al., 2021), for malaria treatment (Barimah, 2013; Boadu & Asase, 2017), research on TM in managing diabetes (Kasilo et al., 2013). Kwame (2021) cites Kwame (2021) as observing that in Ghana, Mali, Nigeria and Zambia, herbal medicine is the first choice for remedying 60% of children with a malaria-induced high fever. This PH focus was emphasised by a team of experts from the Economic Community of West African States (ECOWAS). Most states this systematic review (SR) covers met in 2009 to produce training modules on six priority diseases: Human immunodeficiency virus/Acquired immunodeficiency syndrome, tuberculosis, sickle-cell anaemia, malaria, diabetes and hypertension (all physical health aliments) for TM and MM practitioners (Busia & Kasilo, 2010, p. 20).

In terms of a disparate discussion on integration, various authors have contributed to the discussions on TM and integration in general; however, in the case of MHS, various aspects of integration are covered from diverse perspectives. For instance, integration is sometimes discussed as collaboration (Ae-Ngibise et al., 2010; Arias et al., 2016; Osafo, 2016; van der Watt et al., 2017; Kpobi & Swartz, 2018; Read, 2019; Nyame et al., 2021). In some cases it is seen as partnerships (Nyame et al., 2021). An obstacle to integration is observed as the different conceptualisation of mental health by TM and MM practitioners, thus leading to disagreements among stakeholders (Lambert et al., 2020). These disagreements among stakeholders are in the context of the different ways TM are framed, which leads to different diagnosing and treatment protocols by TM and MM systems (van der Watt et al., 2017; Esan et al., 2018; Nartey et al., 2019; Read, 2019; Gureje et al., 2020; Nyame et al., 2021). There is also mistrust among TM and MM practitioners (van der Watt et al., 2017), with some MM practitioners noting that TM was inferior to MM (Asante & Avornyo, 2013). This leads to TM and MM operating as parallel health systems rather than integrated (Yarney et al., 2013; Opoku et al., 2015; Boateng et al., 2016). Others also addressed the perspectives of key stakeholders on integrating traditional healers of mental illness and MM at the community level in northern Ghana, with inadequate political commitment noted as one key challenge to integration (Yaro, 2016). There were also discussions on the possibilities of collaboration between TM and MM, and the barriers to such collaboration. The obstacles to collaboration observed, included issues of policy, educational support and financial incentives (Badu, Mitchell & O’Brien, 2019).

In terms of health system integration models, different frameworks can be used for discussing integration, such as the framework of complementary-alternative medicine (Lin et al., 2014) and the input, process and output framework (Ahenkan et al., 2019). However, all these frameworks failed to clearly outline the scope, types and levels of integration and enablers of integration, which the RMIC clearly does (Valentijn et al., 2015). The RMIC is therefore proposed and used as an effective model for addressing factors impacting the integration of healthcare systems. These discussions above have highlighted gaps in the literature on the integration of TM and MM in Africa, and therefore in West Africa (WA). These discussions are lopsided in favour of issues that impact TM and MM integration in the PHS. There is therefore a gap in knowledge that needs addressing about the enablers and obstacles to the integration of TM and MM in MHS in WA. In addition, there is an absence of a comprehensive health system integration model or framework used to account for integration in MHS in WA.

The aim therefore, of this SR is to explore the enablers and obstacles (factors) to the integration of TM and MM in MHS in WA using the RMIC. The RMIC is proposed as a useful model of health systems integration because it addresses how all parts of a health system combine with each other to enable or hinder integration (Valentijn et al., 2015). To the best of our knowledge, such a study using the RMIC has never been done.

### The Rainbow Model of Integrated Care

The RMIC is advanced is a model that organises and delivers healthcare in an integrated and holistic way by joining all parts of the health system, which are grouped into: scope (person-focused and population-based), types (clinical, professional, organisational and system) and enablers (functional and normative). An integrated health system can be defined as policy-making, managing, planning, resourcing, budgeting, funding and coordinating of activities to deliver care services so that SUs obtain their required care on a continuum of care services, as and when they require such services over time and across the diverse parts of the health system (WHO, 2008). All integration activities in the health system operate across RMIC’s three levels of the health system: macro, meso and micro levels, with their integration types at each level, such as: macro level (system integration), meso level (professional integration and organisational integration), and micro level (clinical integration). Therefore, integration can either be hindered or facilitated depending on how smoothly operations are aligned and coordinated across the macro, meso and micro levels (Valentijn et al., 2015). For instance, at the:

#### Macro Level (System Integration)

It is at this layer of the health system where system integration occurs. This level can also be defined as the governance section of the health system (Hébert et al., 2008), and seeks to enhance efficiency to ensure that good care is provided to satisfy the requirements of SUs (Kodner, 2009; Suter et al., 2009). System integration connects organisations and aids sharing of resources, essential planning and development (Rosen & Ham, 2008). It at this level where policy-making, orientations and resource allocation take place (Hébert et al., 2008), and outcomes are distributed to the other layers of the healthcare system.

#### Meso Level

At the meso level, two integration types are apparent. These are professional integration and organisational integration. This level can also be termed as the management layer of the health system (Hébert et al., 2008).

#### Professional Integration

Professional integration relates to staff relationships or workings which are intra (inside) and inter (outside) (Kodner, 2009). In some ways, professional integration seeks the incorporation of group practices or essential working among health professionals (Delnoij et al., 2002).

#### Organisational Integration

Organisational integration highlights the relationships or partnerships between healthcare providers or organisations (Kodner, 2009). It describes the extent to which combined working, contracting or essential partnerships are made among healthcare organisations and leads to the sharing of diverse skills and resources together from different organisations or groups to achieve the health needs of SUs (Delnoij et al., 2002).

#### Micro Level (Clinical Integration)

Clinical or ‘service’ integration is seen as the ‘coordination of services and the integration of care into a single process across time, place and discipline’ (Kodner, 2009, p. 11). Clinical integration also means a stable ongoing co-operation and logical links in the primary ways of care provision to SUs (Delnoij et al., 2002, p. 2). Clinical integration is also seen as the operational level of the health system and it is person-focused care, where the efforts of a multidisciplinary team of professionals evaluate and deliver the healthcare needs of SUs (Hébert et al., 2008).

The RMIC also has two enablers of integration. These are functional and normative integrations. Those two play the role of linking up the whole network of integrating parts (Valentijn, 2015; Valentijn et al., 2013, 2015, 2016).

### Functional Integration: Enabler

Functional integration can be explained as how key supporting events, such as financing, human resources, essential planning, information management, and quality improvement are managed throughout the working parts of an organisation to contribute the best to the system of care (Shortell et al., 1996).

### Normative Integration: Enabler

Normative integration, on the other hand, presents a common platform for action across the health system (Valentijn et al., 2013). This common platform advances and preserves a common reference point (that is shared mission, vision, values and culture) between organisations, professional bodies and individuals (Valentijn et al., 2013, p. 30).

Though the AU and WHO have been strong advocates of integrating TM and MM systems in Africa, they also noted challenges that needed addressing to facilitate integration. These challenges included: the little political will for integration, inadequate funding for research on TM, inadequate education and training for TM providers, and insufficient avenues for controlling and regulating safe herbal products (WHO, 2019, p. 57). When these challenges are aligned and synthesised using the RMIC, though the little political will and inadequate funding are initiated at the macro level, they impact integration at all the other levels, because inadequate political will means there are no directives to instruct integration at the meso and micro levels, thus presents cases of inadequate education and training and insufficient avenues for controlling and regulating safe herbal products. Though operationalised at the meso level, the above activities are not coordinated with the relevant professionals and organisations, as such cannot instruct on the provision of safe herbal products to SUs at the micro level. Therefore, RMIC helps to shed light on the factors impacting the integration of TM and MM in MHS in WA. This study will therefore serve as a useful source of knowledge for policy-makers, health professionals and interested stakeholders, which it is hoped will trigger the appropriate responses on issues regarding the integration of TM and MM in MHS in WA.

## Methods

### Research Design

This study was SR conducted in accordance with the Preferred Reporting Items for Systematic reviews and Meta-analyses [PRISMA] (Page et al., 2021), using the relevant parts for the study, such as: inclusion/exclusion criteria, search method, quality appraisal and results. No meta-analysis was done due to the fact that the included studies did not yield to a meta-analysis. A thematic analysis using the RMIC was done to synthesise the findings from the study. The SR question used for this study was: What are the enablers and obstacles to the integration of traditional medicine and mainstream medicine in mental health services in West Africa?

### Inclusion/Exclusion Criteria

The inclusion criteria are as follows: Only English language articles; coverage: articles covering integration and mental health services in any one or more of the 17 listed West African countries; search range: covered 1st January 2001 to 31st December 2021. It is over 20 years (2001) since the WHO and AU rolled out a comprehensive programme for promoting, development and integrating TM and MM. This duration is long enough for some meaningful debates to have taken place to give some indications of enablers and obstacles to the integration of TM and MM integration in West Africa.

### Search Method

Systematic searches were carried out in electronic resources using the free search terms and medical subject headings (MESH). The free terms were used with their equivalent MESH, to ensure comprehensive searches of the databases. Free terms such as ‘traditional medicine’ were used together with MESH for West African countries. Other sources were also searched. These were: the reference lists of Badu et al. (2019) (see Online Table 1). The searches were done between 29th October 2022 and 22nd November 2022, and updated between 24th June 2023 and 30th July 2023, to ensure some publications were not missed. The electronic resources that yielded meaningful results were from: PubMed; PubMed Central; Medline; CINAHL Complete; APA PsycInfo; Web of Science and SocINDEX.

**Fig. 1:**
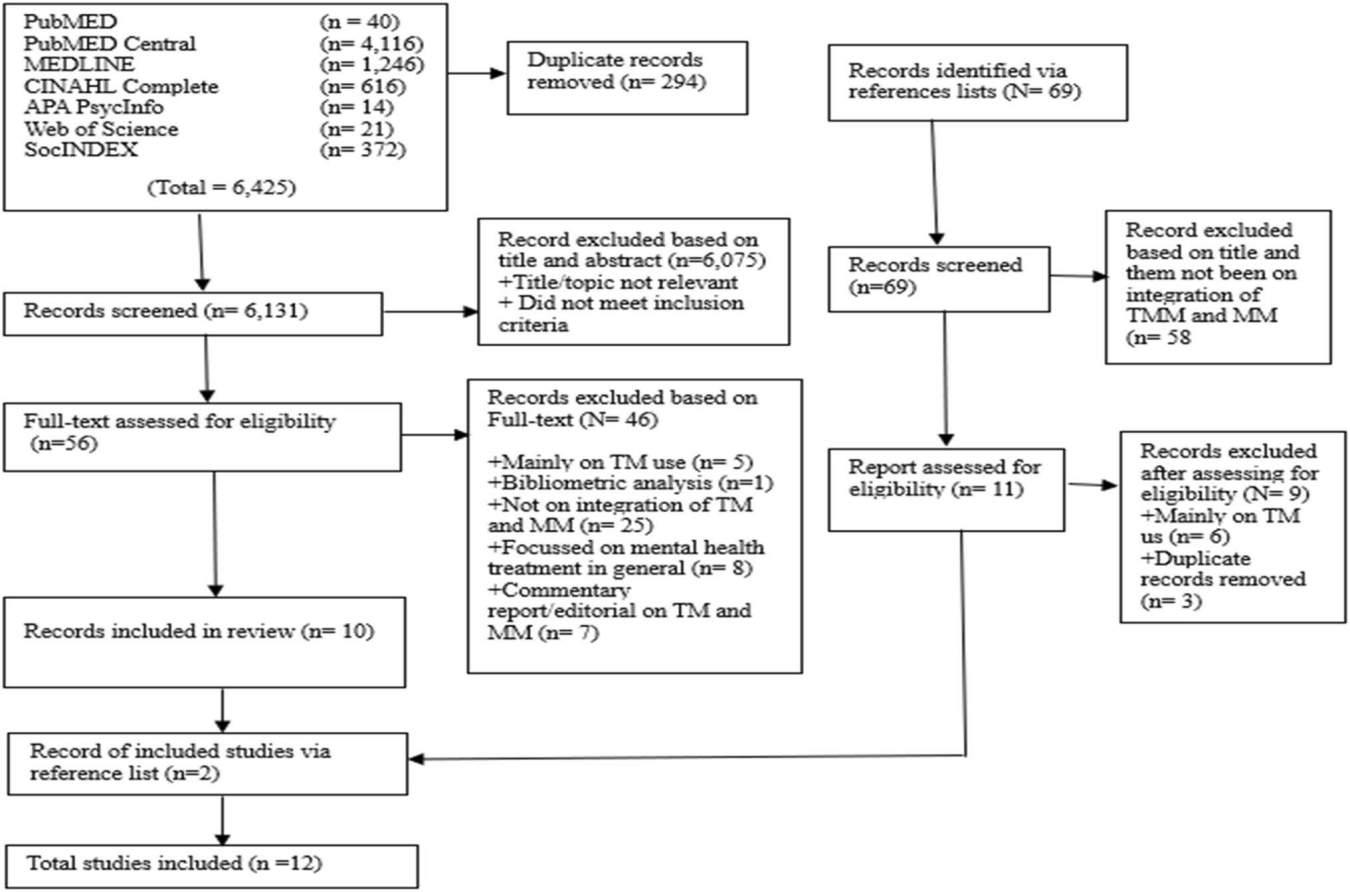
Flowchart of search results. PRISMA diagram adapted from Page et al. (2021)

From the searches (see Fig. 1), a total of 6425 hits were made on articles in the electronic database searches. However, evaluating them using the eligibility criteria, 55 articles from 65 articles were removed after they were identified and assessed for eligibility- after full-text screening. The 55 articles removed were for reasons including: some articles were mainly on TM, or mainly mental health treatment in general, and articles considered areas outside WA. This resulted in 10 studies. Also, the reference list of Badu et al. (2019) was screened, and two studies included. The article (Badu et al., 2019) was a useful source because it covered a similar study, but it was not a SR.

### Quality Appraisal

A move toward the use of evidence-based practice in healthcare decision-making has necessitated the increasing use of systematic reviews. A positive outcome is that reviews of literature can summarise large amounts of research evidence, thus making findings easily accessible (Hawker et al., 2002). This therefore calls for some kind of assessment of the quality of studies selected. Systematic reviews can be a useful resource, however for them to be creditable, the reviewers should be transparent, and should give suitable reason for the SR, such as what was done and their findings (Page et al., 2021). This therefore requires quality appraisal of the studies included.

To facilitate the critical appraisal of the articles included in this SR, the quality assessment tool of studies, known as the Mixed Methods Appraisal Tool version 2018 (MMAT V 2018) (Hong et al., 2018) was used. This is a helpful tool for assessing the methodological quality of studies according to the different designs included in SRs (Crowe & Sheppard, 2011; Rodríguez-Abad et al., 2021). The MMAT V was found to be suitable for qualitative, quantitative and randomised controlled trials (RCTs), and was adopted as it guaranteed the rigorous selection of the articles , to better understand the quality and designs of the studies. The aim of this appraisal was to assess which articles could be included in the review. There is no universal standard of scoring method for heterogeneous data (Hawker et al., 2002). ‘It is discouraged to calculate an overall score from the ratings of each criterion for the MMAT. Instead, it is advised to provide a more detailed presentation of the ratings of each criterion to better inform the quality of the included studies’ (Hong et al., 2018, p. 1). As such studies included were not scored, to ensure the review process encompassed the variety of viewpoints and research methods while remaining transparent and rigorous, and also demonstrating reliability, there should be modification of the review process and also the reporting of results (Hawker et al., 2002). This thinking therefore led to the inclusion of the variety of design categories (qualitative, quantitative and randomised controlled trial) that answered the review question. This variety provided a useful insight to answering the study question. The design categories were therefore screened using their respective five questions in their various categories within the MMAT tool.

Generally, though all the included studies were of good quality (see Online Tables 2–4) when screened with the MMAT tool, some studies had other limitations, which the MMAT tool does not make allowance for explaining. For instance, no questionnaires or interview guides were provided to give an idea of the nature of the questions posed in some studies (Ae-Ngibise et al., 2010; Arias et al., 2016; Esan et al., 2018; Gureje et al., 2020; Read, 2019; Read et al., 2009; Nyame et al., 2021). Biases were not clearly indicated, and when they were indicated, they did not say how they could be reduced in these cases (Ae-Ngibise et al., 2010; Arias et al., 2016; Esan et al., 2018; Ofori-Atta et al., 2018; Read, 2019; Read et al., 2009).

### Data Analysis

A thematic analysis framework was used to derive the themes after the application of PRISMA. Following this, Valentijn’s (2015) RMIC was used to synthesis the findings of the enablers and obstacles to the integration of TM and MM in MHS in WA. A merit there, is that the SR is a comprehensive plan with a search strategy (developed a priori), with the target of minimising bias through identifying, appraising, and synthesising all relevant studies (Zaccagnini & Li, 2023) on TM and MM integration in MHS in WA. It is hoped this will provide useful inputs for policy-making and implementation, thus presenting a better understanding of TM and MM integration. However, caution will have to be exercised when interpreting and generalising the findings, given that there were very few studies on WA and also the SR did not have an extensive coverage of all the states in WA with regard to integrating TM and MM in MHS in WA. The review of studies included was done by all three authors independently before data was extracted using NVivo software.

## Results

The total articles included in the study were 12. These articles were of qualitative, quantitative and randomised controlled trial designs. Qualitative designs were used by nine studies, and the methods used included longitudinal anthropological study/ethnographic methods (Read et al., 2009); qualitative study-situation analysis (Ae-Ngibise et al., 2010); qualitative key informant interviews of literature (Monteiro et al., 2014); comparative method for qualitative data analysis (Arias et al., 2016); thematic analysis (van der Watt et al., 2017); a qualitative study with interviews (Herman et al., 2018); a qualitative formative study (Esan et al., 2018); ethnographic research (Read, 2019); a qualitative cross-sectional survey (Nyame et al., 2021). There was one quantitative study (Nartey et al., 2019), and two RCT studies, one from Ofori-Atta et al. (2018), and a cluster RCT from Gureje et al. (2020). In Online Tables 5 and 6 are the data extracted from the studies included in this review.

### Findings

The aim of this study was to explore the enablers and obstacles to the integration of TM and MM in MHS in WA using the RMIC. This was achieved by bringing together findings from the included studies to come to a conclusion based on the given evidence (Popay et al., 2006). The syntheses of the 12 included studies yielded four themes each under the enablers and obstacles to TM and MM integration in MHS in West Africa. These enablers were: theme 1: Policy and implementation; theme 2: different conceptualisation of mental health; theme 3: trust issues; and theme 4: education and training. The obstacles were theme 1: Policy and implementation; theme 2: referrals; theme 3: trust issues; and theme 4: education and training. These themes either worked as enablers or obstacles of integration of TM and MM, depending on how they were aligned, planned, coordinated, experienced and articulated by stakeholders. These findings are therefore presented as obstacles or enablers of integration within the RMIC at the macro, meso and micro levels, with their corresponding types of integration (system, professional and organisational, clinical).

### Traditional Medicine and Mainstream Medicine Integration in Mental Health Services in West Africa

#### Obstacles to Integration

##### Policy and Implementation Issues: Macro Level

Policy-making takes place at the macro level and it is where systematic integration is initiated. The theme of policy is fundamental as it can work as an obstacle or enabler of integration, and therefore impacts all aspects of MHS directly or indirectly. For instance, policy determines the functions of the themes of: the different conceptualisation of mental health; education and training; referrals; and policy implementation itself. However, the various themes will be discussed in their own merit for clarity. Therefore, a major obstacle to integration was the absence or the inadequacy of policy on integration of TM and MM in MHS in WA (Ae-Ngibise et al., 2010; Arias et al., 2016; Monteiro et al., 2014; Read et al., 2009; van der Watt et al., 2017; Esan et al., 2018; Herman et al., 2018; Nartey et al., 2019; Read, 2019; Gureje et al., 2020; Nyame et al., 2021). This is because without policies clearly spelling out what directives and regulations should be in place and how they should be planned and coordinated, and implemented across the three levels, it can lead to difficulties in integrating the various parts of the TM and MM systems, because stakeholders do not have guidelines on how all integration activities should be structured and implemented. For instance, given that there is no policy on TM and MM integration, it thus makes it difficult for healthcare providers and professionals to determine the type of TM to integrate, given that TM covers a vast array of TM, such as herbal products, divination and prayer camps (faith healing) (WHO, 2013, 2019). Without policy, health providers do not see integration as a priority, and without policy-makers working with and instructing the other levels – meso and micro (health staff and SUs) on which aspects of TM to integrate and how they should be done by outlining the agencies or organisations to be involved, stakeholders are handicapped. Therefore, with the absence of policy, it thus points to the fact that there will be no funding allocated to support integration activities.

##### Different Philosophies or Conceptualisation of Mental Health: Meso and Micro Levels

A major obstacle to TM and MM integration in MHS in West Africa were the difference in conceptualisation of mental health. Though this theme operated across all the three levels and types of integration. It was at the professional and organisational, and clinical integrations strata that the theme was problematic – for instance, healthcare settings and professionals, and SUs did not have a common meaning of mental health to work from. This led to different approaches to diagnosing and treatment, issues with human rights and safety of SUs, and referrals by TM and MM staff and other stakeholders (Ae-Ngibise et al., 2010; Arias et al., 2016; Esan et al., 2018; Gureje et al., 2020; Monteiro et al., 2014; Nartey et al., 2019; Nyame et al., 2021; Read, 2019; Read et al., 2009; van der Watt et al., 2017). For instance, some TM staff and SUs accounted for mental disorders from a supernatural perspective, which MM staff did not condone (Ae-Ngibise et al., 2010). Also, due to the way mental health was seen by TM practitioners, they sometimes adopted unsafe therapeutic procedures leading to human rights abuses. For instance, forcing SUs to fast or shackling them with chains when they were agitated (Ofori-Atta et al., 2018).

##### Trust Issues: Meso and Micro Levels

Trust issues between stakeholders, especially staff from the two systems, were obstacles to integrating TM and MM in MHS. The issue of trust or distrust was noted by several studies (Ae-Ngibise et al., 2010; Arias et al., 2016; Herman et al., 2018; Nyame et al., 2021; van der Watt et al., 2017). Collaboration and partnerships between TM and MM can arguably only best thrive in an atmosphere of trust between healthcare providers from the two systems, and between all other stakeholders. Thus, trust issues among professionals, organisations and SUs was a hindrance to collaborative working among stakeholders.

##### Education and Training: Meso Level

The theme of education and training was mostly evident at the meso level (professional and organisational). An obstacle to integration was the lack or insufficiency of education and training for the smooth incorporation of group practices of TM and MM staff in MHS in WA (Esan et al., 2018). This kept most staff isolated and prevented effective working between the two integrating systems (Monteiro et al., 2014). Though education and training start with policy creation at the macro level, it is at the meso level through intra and inter working of TM and MM professionals and their organisations that are responsible for the implementation of education and training activities in conjunction with educational institutions. Thus due the lack of education and training, there was the lack of the know-how to coordinate and plan towards integration.

#### Enablers of Integration

##### Policy and Implementation: Macro Level

A critical enabler of integration is the creation of policies on integration, delineating all the activities needed for integration. Therefore, systemic integration of policy at the macro level that is linked with professional, organisational, and clinical integrations is essential in ensuring planning and coordinating integration activities across the whole health system and setting up the template for the strategic overview, and the functions of all integrating parts of TM and MM in MHS. Policy framing and their implementation (workings) are invariably the invisible hand that dictates the operations of any health system (Valentijn, 2015), such as outlining the types of TM stakeholder preferences or choices, be they spiritual or physical health therapies (Tabi et al., 2006).

##### Referral System: Meso Level

Having a formal referral system between TM and MM in MHS is a good enabler of TM and MM integration in MHS. This can be viewed in the context of the use of the different approaches to treatment by TM and MM in their provision of more holistic care and strengthening mental healthcare delivery. In some cases, there existed good informal referral systems where TM providers sent SUs to either undergo laboratory tests to better inform TM practitioners’ diagnoses, or in cases when TM treatments did not give positive outcomes for SUs (Herman et al., 2018). For instance, cross-referral between TM and MM in the PHS was noted to yield better health delivery (Gyasi, 2011). This referral relationship can be built on to aid integration, through policy creation of formal referrals channels, and also providing funding and, education and training for stakeholders about this referral system.

##### Trust: Meso and Micro Levels

Integration efforts arguably can best thrive in an atmosphere of trust and cordial working between healthcare providers from the two systems (Ae-Ngibise et al., 2010; Arias et al., 2016; Herman et al., 2018; Nyame et al., 2021; van der Watt et al., 2017;). Trust issues between stakeholders, especially staff from the two systems, was an obstacle to integrating TM and MM in MHS needing to be addressed. Therefore, if stakeholders from TM and MM upheld and respected the perspectives of each other’s system, then trust could be engendered (Ofori-Atta et al., 2018; Read, 2019).

##### Education and Training: Meso Level

Education and training as a facilitator of integration came up pertaining to the smooth working of TM providers and MM providers in MHS (Esan et al., 2018). Skills and competencies acquisition by the relevant professionals from TM and MM in MHS helps to expose relevant staff to what goes on in each other’s system, thereby creating better understanding (Osafo, 2016). These skills and competencies can be provided through tertiary institutions, in-service training and the mass media. Education and training is therefore essential for integration as they enlighten stakeholders about activities and processes needed for integration, such as the policies in place and how they should be implemented.

## Discussion

To the best of our knowledge, this is the first SR conducted on obstacles and enablers of TM and MM integration in MHS in West Africa using the RMIC. The RMIC advances the notion that the operations of these themes at the macro, meso and micro levels can impact the effectiveness of TM and MM integration. This means the integration of activities of stakeholders at the system, professional, organisational and clinical levels of integration are vital, because they have to be planned and linked up on a continuum of care services across the diverse parts of the health system to provide the SUs with the required healthcare (WHO, 2008).

### Implications of Enablers and Obstacles on Integration

#### Policy and Implementation

It is worth indicating that policy alone at macro level does not lead to integration. Policies must be worked out and coordinated across all parts of the health system and to a large extent define the types and levels of integrations. Therefore, the creation of polices and attempts to implement them without linking them to the other two levels, or just placing TM services in MM services, does not constitute integration (Yaro, 2016). Thus, a key obstacle to integration was the incoherence or absence of policy and poor government policy implementation from the macro level, where systematic integration occurred. For instance, the inconsistent or lack of interest in the implementation of policies on integrating mental health and primary healthcare in Uganda and Ghana have been noted as one of the obstacles to integration (Bhana et al., 2010). This is because poor and ineffective policies do not inspire health professionals, organisation and SUs to work together. For instance, the theme of policy, its poor implementation, or the absence of policy on aspects of integrating TM and MM in MHS had a negative impact on all the other four themes. In this case, systematic integration operations had no clearly defined ways of connecting with organisational and professional integrations activities. There were no formal policy fine-tuning channels between policy-makers, health managers and other health professionals, and SUs to actively engage to iron out difference or misunderstandings. This is critical, as these channels update and inform various stakeholders as to what was possible and their impacts.

Just as incoherent, poor or ineffective government policy and implementation was a major bane to the integration, this can also be a critical enabler in serving as a linchpin in facilitating other factors for the integration of TM and MM in MHS in West Africa. For instance, systematic integration levels activities ensure that there are clear policies in place on all aspects on integration and how they link with the meso and micro level integration activities to clarify expectations, behaviour and actions of all staff, SUs, the government and all stakeholders. Therefore, along this line, there need to be alliances and knowledge networks among TM and MM in MHS, together with the sharing of competences, responsibilities and accountabilities, which are facilitated by tapping into common human resources, planning together, information management (functional integration), and sharing a mission, vision and values (normative integrations). These two enablers safeguard the smooth working together of TM and MM systems by enabling shared activities, such as financial, management and information systems being built around the core processes of service delivery to coordinate and support accountability and decision-making across the health systems (Valentijn, 2015). In short, this means system integration actions must also be aligned and coordinated at the meso level, with organisational and professional plans linked to micro level actions, ensuring that clinically, SUs' health needs are properly responded to through correct planning, alignment and coordination of health activities to achieve the desired outcomes expected by SUs.

#### Different Conceptualisation of Mental Health

A major barrier to integration was an absence of a fixed conceptualisation of mental health and common treatment pathways from TM and MM perspectives, set at the systematic level integration. This was problematic, because without a fixed reference point, situated and linked to clinical, professional and organisational activities, and enabled by functional and normative integration processes, meant integration activities were not coordinated. For example, because there are hardly any policies to work from as a common reference point on how mental health should be seen by TM and MM staff, there were various approaches to diagnosis and treatment, therefore making it difficult for integration, thus leaving the two systems to function as parallel systems. This resulted in a thinking of, “we have what the doctors do and we have what the pastors do” as noted in one study (Read, 2019, p. 624). For example, MHS professionals in MM systems were usually sceptical about the diagnosing and treatment claims of TM providers, because of their reliance on spiritual entities (Arias et al., 2016). This obstacle to integration can be viewed from the diverse understanding of TM and MM staff and the rest of the community’s attitudes to mental health disorders. For instance, people in the community sometimes have a different accounting for mental illness, which is cultural and spiritual in nature (Monteiro et al., 2014). Though some attribute mental illness to physical health or organic sources, like substance abuse—cannabis, cocaine or alcohol—most MM mental health providers saw mental illness as originating from ‘physical sources’ and not spiritual (Arias et al., 2016). This dichotomy is exemplified by a pastor’s noting that “It is the spirit that asks them to do it. You tell the spirit to stop, that you will not do this again, but you can't. Unless you add prayer, you don't have the strength to overcome the thing” (Arias et al., 2016, p. 6). This means physical health issues are treated by physicians, and the spiritual issues are treated by priests or pastors (Osafo et al., 2015; Read et al., 2009). Due to the difference in the way health is seen by TM and MM providers, there are no systemic, organisational and professional integration activities across all the levels of the TM and MM systems. There are no formalised templates for back-and-forth referral systems between TM professionals and MM professionals, therefore, MHS referrals efforts are informal and based on personally forged connections (Herman et al., 2018; Nyame et al., 2021; Read, 2019). This also leads to poor human rights and unsafe conditions SUs are exposed to in TM settings. Human rights may be viewed as grounded within a moral code, which promotes social welfare, solidarity, and harmony in human relationships, rather than legalistic frameworks by some in the community (Read et al., 2009) based on macro, meso and micro level agreements and considerations by TM and MM providers of MHS. Therefore, these ad hoc arrangements cause uncertainty in the lines of authority in terms of practice between TM and MM professionals, especially from the TM providers’ view, which was one of the reasons TM staff were declining to collaborate (Read, 2019).

It therefore stands to reason that against the background of these difficulties, policies and effective guidelines could be provided on agreed codes of practice and operations on ‘all’ contested areas between TM and MM providers such as policy and implementation, diagnosing, human rights and safety of SUs. However, the key to the effectiveness of the above operations will depend on training and education. Training and education should encapsulate professional and organisational integration activities, linked to systemic integration, decision-making and service provision for SUs at the clinical integration level, which upholds and supports TM and MM providers’ collaboration. The RMIC therefore allows for the factoring into systemic integration by policy-makers, professional integration and organisational integration by health professionals and the organisations in TM and MM systems, such as the different conceptualising of mental health and the different cultural attitudes to effective health provision (Broom & Doron, 2013). This is because how people see health is interwoven with their complex social relations (Del Casino, 2004), and without understanding this, even the best healthcare system will not be able to meet the needs of some SUs. This means clinical integration level expectations of SU must be channelled all the way to macro level from all the other levels in a back-and-forth manner. For instance, if the community feels mental illnesses has supernatural origins, this must be addressed—not ignored—at all levels of integration (Kyei et al., 2012). Policies could clearly set out areas of divergence and where collaboration could be strengthened to enhance integration. This is significant, in that it provides common grounds for collaboration; therefore, avoiding the confrontational ways, as advocated by some human rights-based agencies for addressing abuses, unsafe and unethical practices in TM settings (Read, 2019). For instance, some MM staff noted there should be boundaries which TM and MM agree to work within, with each system knowing and understanding their limits, thus knowing when to make a referral when the mental health disorder is out of their competencies (van der Watt et al., 2017). The adherence to referral and other shared polices promotes adherence to treatment regimens and mitigates against some of the inhumane practices, such as forcing SUs who are already ill to fast (Arias et al., 2016). This means system integration level activities should be planned and aligned with organisational, professional and clinical level integration activities to enable linkages. Therefore, coordinating TM providers’ work and nurturing good relationships across the levels enables collaboration without causing threats between MM and TM professionals, therefore aiding TM and MM integration (Read, 2019).

#### Trust Issues

Trust issues among TM and MM providers in MHS levels undermined integration (Ae-Ngibise et al., 2010; Arias et al., 2016; Herman et al., 2018; Nyame et al., 2021; van der Watt et al., 2017). The attitudes of MM providers looking down and disengaging with their TM counterparts was a major problem (Wreford, 2005). This was due to the fact that professional integration of TM and MM staff and their organisations had very few working relationships. For an illustration, in Ghana, MM mental health services are seen as superior, widespread, better supported and promoted by the government to the detriment of TM; there are therefore conflicts and tensions between TM and MM professionals due to lack of mutual understanding of each other’s systems (Osafo, 2016), with TM providers feeling they are not appreciated by MHS (van der Watt et al., 2017). This was due to macro and meso level activities not being planned and coordinated on issues such as diagnosing, medication prescriptions and referrals. This does not foster trust and co-operation between TM and MM providers. Some sus at the micro level also expressed a distrust of meso level MM mental health staff and their ability to treat mental illnesses, due to the fact that some MM professionals dismissed TM practices. This was compounded by the poorly defined roles and responsibilities of TM providers in MM mental health settings, thus resulting in distrust, snobbery, rivalry and perceptions of superiority (van der Watt et al., 2017). Trust-building is an essential enabler in the integration of TM and MM in MHS in WA. With professional group collaboration at all levels of TM and MM, staff can deepen and strengthen ties between TM and MM in MHS. For instance, some form of informal referral relationships existed with some MM (hospitals and psychiatric units) due to trust, where TM providers signposted SUs to MM facilities for physical medical evaluation before they were treated (Arias et al., 2016) or referred for treatment, or to undergo laboratory investigation or tests to help TM providers in their diagnoses, or when their therapies were not producing good results (Herman et al., 2018). These overtures should be capitalised on and strengthened to support integration.

#### Education and Training

Poor policy input has meant educational and training activities to aid integration are absent or are half-hearted, thus working as an obstacle to integration (Esan et al., 2018). Although education and training policies are initiated at the governance level of the health system, it is at the professional integration and organisation integration levels that they come into full effect. Therefore, if educational and training polices are not linked with staff and their organisational activities, then they will be ineffective. For example, in Senegal some politicians saw the integration of TM and MM as a way to ease the burden on hospitals. However, MM medical doctors protested against TM, as allowing witchcraft into the formal health sector (Haque, 2018). This clearly demonstrated the fact that at the macro level, policy and health provision activities were not coordinated with the meso and micro levels work, with little or no education and training on TM for staff, hence the ignorance displayed by the medical doctors. This was critical because these medical doctors did not share common values or missions with TM practitioners, let alone communicated and supported them. Notwithstanding this, a strong positive enabler of integration is education and training. This can be done by incorporating TM curricula in medical, nursing, pharmacy institutions. This reduces the alienation of MM mental health professionals from TM and TM professionals. Some aspects of this have been done in Ghana and Nigeria (WHO, 2002); also, education and training activities in Kenya on the integrating of mental health services into existing primary care yielded positive results such as improved therapeutic communication between stakeholders and knowledge of healthcare professionals (Kiima & Jenkins, 2010). Training on integrating mental health and primary care in Nigeria has also shown improvement in the knowledge and skills of the health professionals, in terms of identifying and treating mental, neurological and substance use disorders, and has also facilitated referrals (Gureje et al., 2015). Though there was interest in working with MM providers by TM providers, there were mutual undertones of suspicion between TM and MM professionals (Nyame et al., 2021); education and training can therefore help to address these suspicions (Osafo et al., 2015). Some TM providers also advocated public education and training as a way to foster collaboration (van der Watt et al., 2017) to help address the issues of superiority and tensions, but this must be reflected at the system, organisational professional, and clinical integration levels. For example, the promotion of education, research and referral mechanisms and collaborative working between MM and TM providers have the benefits of helping to integrate the two systems, if integrated across all the levels.

Research linked to education and training activities in the conducting of field research with TM providers have indicated integration ties can be forged by education and research (Ofori-Atta et al., 2018). For instance, TM staff and MM researchers collaborated on a RCT in TM in prayer camps, which yielded positive results. The RCT study of the clinical effectiveness of combining MM in MHS intervention (psychotropic medication) and TM (in the experimental group), with SUs diagnosed with schizophrenia and mood disorders, registered significantly lower rates in the experimental group in terms of symptoms such as thinking disturbance and depression. This study involved 71 SUs in the experimental group and 68 SUs in the control group over six weeks (Ofori-Atta et al., 2018). Also, another study enabled the assessment of the effectiveness and cost-effectiveness of a collaborative shared care model for psychosis delivered by TM providers and MM providers in TM settings for 51 clusters randomly allocated (26 intervention, 25 control) in Ghana and Nigeria. The study noted there was an improvement in participants’ unusual thought content, hallucinatory behaviour, mannerisms and blunted affect (Gureje et al., 2020), meaning that collaborative care between TM and MM MHS staff improved SUs’ health.

### Limitations and Strengths of the Study

A strength of this work is that we believe it is the first of its kind exploring the enablers and obstacles to the integration of MM and TM in the MHS in West Africa using the RMIC. The strength lies in the fact that the three levels of the RMIC outline the various activities that take place in each level, thus allowing for the investigation of integration activities and experiences at the various levels, and how they enable or hinder integration in a holistic way. A limitation of this study was that it was limited to works in the English language. This meant the study did not evenly cover all the West African countries. Also, because 11 states out the 17 countries are French-speaking, while two states are Portuguese-speaking, and one state was both French- and English-speaking, in terms of official languages this meant relevant publications in other languages (French and Portuguese) could have been missed.

## Conclusion

Governments across WA with the support of the WHO have championed the integration of TM and MM in health systems as a way of providing holistic, accessible, affordable and acceptable healthcare. Despite this interest, what was clear from the literature was the large focus on the integration of TM and MM in PHS to the neglect of MHS in WA. This paucity of literature on TM and MM integration in mental health services highlighted the absence of a cohesive framework for accounting for integration. This SR therefore provides all stakeholders and policy-makers with a holistic perspective on integration using the RMIC. This study therefore provided a useful insight on obstacles and enablers of integration of TM and MM in MHS in WA.

This study identified a number of factors that impact TM and MM integration in MHS in West Africa. The RMIC was used to demonstrate that for integration to take place, it must happen across the macro, meso and micro levels, with integration activities linking up across all the levels. It was evident that a key obstacle and enabler of integration was policy (and implementation). This was because policy-making and implementation initiated the process of integration under systemic integration levels, but the absence of policies on the different conceptualisation of mental health, education and training was problematic when linked to professional integration and organisation integration, and clinical integration activities. Activities across the RMIC therefore indicated that a close, back-and-forth engagement of system integration, professional integration and organisation integration, and clinical integration activities promoted the integration of TM and MM in MHS in WA. For instance, absent or poor policy implementation across macro, meso and micro levels of TM and MM MHS, meant there was no coherent policy on what mental health was, the provision of education and training, and therefore, on the implementation of policies. Good policies and their implementation and integration across the macro, meso and micro levels were seen as the great starting enablers of TM and MHS integration in MHS in WA. This was because these policies were seen as the foundation on which other aspects of the health system were built. Therefore, the difficulties with the difference in the perspectives of what mental illness is, from the TM and MM perspectives, could be resolved with the forging of workable frameworks. This can be achieved through education and training. This by and large helps to reduce the perceived competition between TM and MM providers, thereby reducing the mistrust and engendering positive working relations.

The lesson for policy-makers and stakeholders interested in integration is that ad hoc measures, informal initiatives by staff on integration, or situations where TM services are just placed in MM mental health services do not amount to integration. The integration of TM and MM in MHS in WA involves policy-making, planning, management, resourcing and the coordinating of activities from the macro, meso and micro levels to provide care services required by SUs over time and across the diverse parts of the health system. The use and a close adherence to the principles of RMIC can go a long way to support integration of TM and MM in MHS in WA. Therefore, more research is needed on the obstacles and enablers to TM and MM integration in MHS in various West Africa countries, to better evaluate these factors of integration.

### Supplementary Information

Below is the link to the electronic supplementary material.

Supplementary file1 (DOCX 38 KB)

## Abbreviations

AU: African Union
MHS: Mental health services
MM: Mainstream medicine
PANSS: Positive and Negative Syndrome Scale
RMIC: Rainbow model of integrated care
SUs: Service users
TM: Traditional medicine
WA: West Africa
WHO: World Health Organisation
