# Supplementary material for: Factors Influencing the Integration of Traditional Medicine and Mainstream Medicine in Mental Health Services in West Africa: A Systematic Review Using Narrative Synthesis
Source: Community Ment Health J. 2024 Apr 15;60(6):1117–30. doi: 10.1007/s10597-024-01263-w (PMC11199277; doi:10.1007/s10597-024-01263-w)
Supplement: Supplementary file 1 — Supplementary file1 (DOCX 38 KB) [file 10597_2024_1263_MOESM1_ESM.docx]

**Supplements**

**Table 1 Search strategy - keywords and phrases**

| **Search terms**  West Africa [MeSH Terms] OR West Africa OR (Benin OR Burkina Faso OR Cameroon OR Cape Verde OR Côte d’Ivoire OR The Gambia OR Ghana OR Guinea, Guinea Bissau OR Liberia OR Mali OR Mauritania OR Niger OR Nigeria OR Sierra Leone OR Sénégal OR Togo) AND "Traditional medicine" OR "traditional therap*" OR "African traditional medicine" OR "traditional healer" OR "traditional medicine practitioner" OR "traditional medicine healer" OR "traditional birth attendant" OR bonesetter OR "faith healer" OR spiritualist OR "Complementary therap*" OR “Complementary OR alternative medicine" OR "Herbal medicine" OR "herbal therap*" OR "medicinal herbs" OR "herbal product" OR "herbal remedies" OR herbalist OR "medical herbalist" OR "folklore medicine" OR "folklore therapy" OR "indigenous therapy" OR "indigenous medicine" OR "non-allopathic therap*" OR "indigenous health"  AND Mental health* OR Mental illness* OR Mental disorder* OR psychiatr*  AND Integrat* OR “Integrative Medicine” OR Intervention OR collaborat* OR use  AND barriers OR challenges OR hind* OR problems OR obstacles OR challenges |
| --- |

**Summary of Quality Assessment**

**Table 2 Category of study designs: Qualitative**

|  | Author/year | Screening questions | | Qualitative study | | | | |
| --- | --- | --- | --- | --- | --- | --- | --- | --- |
|  |  | Are there clear research questions? | Do the collected data allow one to address the research questions? | Is the qualitative approach appropriate to answer the research question (objectives)? | Are the qualitative data collection methods adequate to address the research question? | Are the findings adequately derived from the data? | Is the interpretation of results sufficiently substantiated by data? | Is there coherence between qualitative data sources, collection, analysis and interpretation? |
| 1 | Read, Adiibokah and Nyame (2009) | Yes | Not fully explained | Yes | Yes | Not fully explained | Yes | Yes |
| 2 | Ae-Ngibise *et al.* (2010) | Yes | Not fully explained | Yes | Yes | Not fully explained | Yes | Yes |
| 3 | Monteiro *et al.* (2014) | Yes | Yes | Yes | Yes | Yes | Yes | Yes |
| 4 | Arias *et al*. (2016) | Yes | Not fully explained | Yes | Yes | Not fully explained | Yes | Yes |
| 5 | van der Watt *et al*. (2017) | Yes | Yes | Yes | Yes | Yes | Yes | Yes |
| 6 | Herman *et al*. (2018) | Yes | Yes | Yes | Yes | Yes | Yes | Yes |
| 7 | Esan *et al*. (2018) | Yes | Not fully explained | Yes | Yes | Not fully explained | Yes | Yes |
| 8 | Read (2019) | Yes | Not fully explained | Yes | Yes | Not fully explained | Yes | Yes |
| 9 | Nyame *et al.* (2021) | Yes | Not fully explained | Yes | Yes | Yes | Yes | Yes |

**Summary of Quality Assessment**

|  | Author/year | Screening questions | | Quantitative descriptive | | | | |
| --- | --- | --- | --- | --- | --- | --- | --- | --- |
|  |  | Are there clear research questions? | Do the collected data allow one to address the research questions? | Is the sampling strategy relevant to address the research question? | Is the sample representative of the target population? | Are the measurements appropriate? | Is the risk of non-response bias low? | Is the statistical analysis appropriate to answer the research question? |
| 10 | Nartey *et al*. (2019) | Yes | Yes | Yes | Yes | Yes | Yes | Yes |

**Table 3 Category of study designs: Quantitative**

**Summary of Quality Assessment**

**Table 4 Category of study designs: Quantitative randomised controlled trials**

|  | Author/year | Screening questions | | Quantitative randomised controlled trials | | | | |
| --- | --- | --- | --- | --- | --- | --- | --- | --- |
|  |  | Are there clear research questions? | Do the collected data allow one to address the research questions? | Is randomisation appropriately performed? | Are the groups comparable at baseline? | Are there complete outcome data? | Are outcome assessors blinded to the intervention provided? | Did the participants adhere to the assigned intervention? |
| 11 | Ofori-Atta *et al.* (2018) | Yes | Yes | Yes | Yes | Not fully explained | Yes | Yes |
| 12 | Gureje *et al.* (2020) | Yes | Not fully explained | Yes | Yes | Yes | Yes | Yes |

**Table 5** **Studies included for the review**

| Authors / year / country of study | Study title | Objectives of the study | Study design |
| --- | --- | --- | --- |
| Read, Adiibokah and Nyame (2009)  Ghana | Local suffering and the global discourse of mental health and human rights: An ethnographic study of responses to mental illness in rural Ghana | The paper draws on people with mental illness, their families and healing practitioners within rural communities in Ghana, to gain an understanding of how practices such as the chaining and beating of those with mental illness are embedded within sociocultural meanings and responses evoked by madness or mental illness. | Qualitative study-longitudinal anthropological study / Ethnographic methods. |
| Ae-Ngibise *et al.* (2010)  Ghana | ‘Whether you like it or not people with mental problems are going to go to them’: A qualitative exploration into the widespread use of traditional and faith healers in the provision of mental health care in Ghana | The first aim of this study was to explore the reasons underpinning the widespread appeal of traditional / faith healers in Ghana. This formed a backdrop for the second objective, to identify what barriers or enabling factors that may be there for forming bi-sectoral partnerships. | Qualitative study-situation analysis. |
| Monteiro *et al.* (2014)  Senegal | Policy perspectives and attitudes towards mental health treatment in rural Senegal | The study explores healthcare workers' and policy stakeholders’ knowledge and attitudes regarding mental illness, interactions with patients in the community, and perceived training needs at a health clinic in rural southeastern Senegal. | Qualitative key informant interviews. |
| Arias *et al.* (2016)  Ghana | Prayer camps and biomedical care in Ghana: Is collaboration in mental health care possible? | Sought to examine the beliefs and practices of prayer camp staff and the perspective of bio-  medical care providers, with the goal of characterising interest in and potential for inter-  sectoral partnership between prayer camp staff and biomedical care providers. | Purposive sampling method to recruit participants and  qualitative data analysis. |
| van der Watt *et al*. (2017)  Ghana, Kenya*,  and Nigeria | Collaboration between biomedical and complementary and alternative care providers: barriers and pathways | They examined the scope of collaborative care for persons with mental illness as implemented by traditional healers, faith healers, and biomedical care providers. | Formative studies conducted to inform the design and implementation of a single-blind cluster RCT.  Semi-structured focus group discussions used. |
| Esan *et al*. (2018)  Ghana, Kenya*,  and Nigeria | A survey of traditional and faith healers providing mental health care in three sub-Saharan African countries | The aim of the study was to describe the profile, practices and distribution of traditional and faith healers in three sub-Saharan African countries in great need for major improvements in their mental health systems, namely Ghana, Kenya* and Nigeria. | Formative studies that preceded the launching of a randomised controlled trial. |
| Herman *et al*. (2018)  Liberia | Closing the mental health treatment gap through the collaboration of traditional and Western medicine in Liberia | This paper examines the potential for collaboration between traditional and Western medicine to close the mental health treatment gap in Liberia. | Qualitative study |
| Ofori-Atta *et al.* (2018)  Ghana | Joining psychiatric care and faith healing in a prayer camp in Ghana: randomised trial | To ethically evaluate joining traditional faith healing with psychiatric care including medications. | An open RCT with masked assessments comparing prayer camp care plus psychiatric / psychopharmacologic treatment supported by nurse clinical oversight with prayer camp care alone for a diverse set of medication sensitive conditions. |
| Nartey *et al*. (2019)  Ghana | The predictors of treatment pathways to mental health services among consumers in Ghana | To explore factors influencing treatment pathways to mental health services among consumers in Ghana. | Cross‐sectional design using quantitative method. |
| Read (2019)  Ghana | Rights as Relationships: Collaborating with Faith Healers in Community Mental Health in Ghana | Explores the ways in which mental health workers think through the ethics of working with traditional and faith healers in Ghana. | Ethnographic research |
| Gureje *et al*. (2020)  Ghana,  and Nigeria | Effect of collaborative care between traditional and faith healers and primary health care workers on psychosis outcomes in Nigeria and Ghana (COSIMPO): a cluster randomised controlled trial | Assessed the effectiveness and cost-effectiveness of a collaborative shared care model for psychosis delivered by traditional and faith healers and primary healthcare providers. | Cluster randomised trial |
| Nyame *et al*. (2021)  Ghana | Perceptions of Ghanaian traditional health practitioners, primary health care workers, service users and caregivers regarding collaboration for mental health care | Examines the possibility of forging partnerships at the primary healthcare (PHC) level in two geopolitical regions of Ghana, to maximise the benefits to both health systems. | A qualitative cross-sectional survey. |

* Data on the country not included

**Table 6 Summary of the characteristics included studies**

|  | Author (s) /  year of publication / country of study | Data collection tools | Sample size | Participants sampled | Findings | Limitations |
| --- | --- | --- | --- | --- | --- | --- |
| 1 | Read, Adiibokah and Nyame (2009)  Ghana | Purposive sampling-observing / interviews / participation. | Over 40 homes were visited in addition to the shrine and prayer camps, and a total of 67 participants were interviewed, including patients, carers, three traditional healers, pastors, mallam and imams. Focus group discussions were held including registered mental nurses, young people, Muslims, cannabis users, church members and parents. | Households with a family member with mental illness, as well as churches, shrines, hospitals and clinics. | Withholding of food ('fasting'), chaining and beating of the mentally ill was found to be common.  Responses to mental illness were embedded within spiritual and moral perspectives.  Families struggled to provide care for severely mentally ill relatives with very little support from formal health services.  Psychiatric services were difficult to access, particularly in rural communities.  Traditional and faith healers remained highly popular despite the routine maltreatment of the mentally ill in their facilities. | Most of which are inherent in the anthropological approach with its focus on 'ethnographies of the particular' / in generalising these findings since the sample size is small and particular personal, historical, social and cultural factors will vary.  The use of Twi as the lingua franca may have disadvantaged those for whom it was not their first language, and the process of translation inevitably leads to some loss or distortion of meaning. |
| 2 | Ae-Ngibise *et al.* (2010)  Ghana | Purposive sampling - interviews and focus group discussions. | 81 semi-structured interviews and  seven focus group  discussions were conducted with 120 key stakeholders. | Policy-makers, health professionals, users of psychiatric services, teachers, police officers, academics, and religious (Christian and Islamic) traditional healers. | Barriers to collaboration, including human rights and safety concerns, scepticism around the effectiveness of ‘conventional’ treatments, and traditional healer solidarity were identified. Mutual respect and bi-directional conversations surfaced as the key ingredients for successful partnerships. Promoting greater understanding, rather than maintaining indifferent distances may lead to more successful co-operation in future. | The sample is too small to allow generalisation of the findings on a larger scale. |
| 3 | Monteiro *et al.* (2014)  Senegal | Qualitative key informant interviews. | Eight interviews. | Health workers’ (physicians, nurses, midwives, community health workers) and policy-makers. | Staff encounter many patients with emotional / psychological problems or mental illnesses, and they employ various strategies in treating these patients.  There is a need for more training to address and diagnose mental health problems, especially severe psychiatric illnesses. | The small sample size suggests that additional studies are needed to examine other important topics and generalise these initial findings. |
| 4 | Arias *et al.* (2016)  Ghana | Open-ended, semi-structured interviews | 50 prophets (4), church elders (5), pastors (2), a reverend, a church member, and a caretaker. The hospital sample included registered mental health nurses (29), general practitioners (2), an orderly, a hospital administrator, a psychiatrist, a community mental health officer, and a community psychiatric nurse. | Prophets and staff at nine  Christian prayer camps. | Prayer camp staff interested in collaboration with biomedical mental healthcare providers in technical support and introducing medications.  Prayer camp had shortcomings in their infrastructure and hygienic conditions.  Challenges to collaboration - prayer camp staff expressed strong beliefs in a spiritual rather than biomedical explanatory model for mental illness, used fasting and chain restraints in the course of treatment, endorsed only short-term use of medication to treat mental illness — expressing concerns about long-term medication use.  Biomedical providers were sceptical about the spiritual interpretations of mental illness held by faith healers - concerned by the use of chains, fasting, and the lack of adequate living facilities in prayer camps. Many were, however, interested in engaging with prayer camps to expand access to clinical care for patients residing in the camps. | The study focused on prayer camps in Ghana, thus the results may differ in other settings.  The findings are limited by the possibility that respondents may not have answered questions honestly. Researchers were not able to talk with patients or family caregivers, whose perspectives would be important to the issue and should be included in future studies of potential partnerships between faith healers and medical providers. |
| 5 | van der Watt *et al*. (2017)  Ghana, Kenya*,  and Nigeria | Focus group discussions (FGD) | 25 semi-structured FGDs were conducted in  Ghana (nine), Nigeria (eight).  The Ghanaian sample was (72) and consisted of 15 faith healers (FH), 16 traditional healers, 16 patients and their caregivers, and 25 biomedical care providers.  The Nigerian sample (61) consisted of 14 faith healers, 15 traditional healers, 16 patients and their caregivers, and 16 biomedical care providers. | Faith healers, traditional healers, patients and their caregivers), as well as biomedical care providers were sampled in each country. | Barriers to collaboration: distrust, influenced by factionalism, charlatanism, perceptions of superiority, limited roles, and responsibilities.  Ways to better collaborate: education, formal policy recognition and regulation, and acceptance of mutual responsibility. | Presenting the data from the point of the three nations may have compromised the depth of the data analyses in terms of the perceptions of specific stakeholders or country-specific findings.  The use of FGD can highlight the degree of consensus - yet there is a risk of “group think” and “group polarisation” (Robbins and Judge, 2011). It is thus possible that some participants may have suppressed, withheld, or modified their true feelings and / or beliefs if these were different from the majority in the group. |
| 6 | Esan *et al*. (2018)  Ghana, Kenya*,  and Nigeria | Desk reviews / proforma was used to collect information / direct observation. | 205 CAPs in Ghana and 82 in Nigeria. | Complementary and alternative mental health service providers (CAPs), traditional and faith healers. | TM providers used a combination of herbs, divination and rituals in the treatment of mental disorders.  Use of physical restraints by CAPs to manage patients was relatively common in Nigeria (63.4%) and Ghana (21%).  TM providers have between two- and 10-fold capacity for patient admission compared to conventional mental health facilities. | The inability to verify the claims made by the CAPs.  The snowball sampling technique that was adopted for the study may be impossible to determine sampling bias or make accurate inferences about the CAPs based on the data obtained. |
| 7 | Herman *et al*. (2018)  Liberia | Participant interviews | 35 traditional healers and service users. | Traditional healers and service utilisers | Participants said cultural attitudes, beliefs, and structural factors may influence collaboration between traditional and Western medicine.  Structural factors such as geographic distance and financial barriers made traditional medicine more accessible than Western medicine.  Healers expressed willingness to collaborate in order to strengthen their skills, but Western physicians were hesitant to collaborate.  Liberians believed in both medical traditions, though preferred Western medicine. | The sample was made up of a small number of Liberian traditional healers and service users, indicating that the data may not reflect the opinions of other traditional healers or service users outside the Monrovia metropolitan area.  Additionally, selection bias may have occurred as participating may share the desire to collaborate with Western physicians and expand knowledge on treating mental illness. Social desirability bias may have also occurred due to the presence of American researchers and Liberian medical students throughout the interviews. |
| 8 | Ofori-Atta *et al.* (2018)  Ghana | Card sorting /  24-item Brief Psychotic Rating Scale (BPRS) method. | Randomly assigned to receive either indicated medication for schizophrenia or mood disorders, along with usual prayer camp activities (prayers, chain restraints and fasting) [71].  The prayer camp activities alone  (68). | Residents of a Ghana prayer camp getting treatment. | Total brief psychotic rating scale symptoms of SUs were significantly lower in the  experimental group (P = 0.003, effect size –0.48) than service users who had medication for schizophrenia or mood disorders along with usual prayer camp activities (prayers, chain restraints and fasting). There was no significant difference in days in chains. | First, the study was only for six weeks. For the benefits identified here to translate into durable health improvements, treatment must be sustained past the period of prayer camp involvement through accessible out-patient services and a reliable medication supply.  Secondly, the trial presented here was supported by a special allocation of medical and support staff and of medication. These resources, although accessible in principle, are not always available in Ghana. Sustained benefits ultimately depend on the availability of a broad range of both trained health professionals and prayer camp staff who are willing and eager to collaborate. |
| 9 | Nartey *et al*. (2019)  Ghana | Questionnaires. | 542 mental health services users. | Mental health services users from five health facilities. | Treatment ways for mental illness were general hospitals / clinics, psychiatric hospitals, and faith‐based practices.  Age, household size, primary occupation, ethnicity, marital status, religion, and geographic location, as well as attitudes  and beliefs, affordability and severity of mental illness were significant predictors of treatment pathways. | The study recruited service users accessing outpatients’ mental health services in the hospital setting and their caregivers, without the perspectives of health professionals and health systems planners. The SUs coming to the hospital are probably more likely to subscribe to a biomedical model of mental illness, and a conventional orthodox medicine treatment model. This perception could possibly influence their response to the questions on treatment pathways and so affect the validity of the strength of the associations drawn in the conclusion. |
| 10 | Read (2019)  Ghana | Conversations, interviews and FGD. | Not specified. | Conversations, interviews and FGD were conducted with persons who had been diagnosed with mental illness, family members, members of the public, healers, health workers and other relevant actors, such as workers for NGOs and humanitarian organisations. | Mental health workers attempted to negotiate the tensions between their professional duty of care, their Christian faith, and the authority of healers.  Rather than enforcing legal prohibitions, mental health workers sought to avoid confrontation and manoeuvre within existing hierarchies, thereby preserving sentiments of obligation and reciprocity within a shared moral landscape and established forms of sociality. | The study could be open to selection bias, because the choice of what was relevant was from the writer’s point of reference. |
| 11 | Gureje *et al*. (2020)  Ghana,  and Nigeria | Face-to-face interviews. | A cluster consisted of one primary care clinic (PHC) and all the traditional and faith healers (TFH) facilities in the catchment area served by the PHC. A cluster was thus composed of one PHC and between one and five TFH facilities. Across the two sites, a total of 71 clusters were formed following this procedure (37 in Ghana and 34 in Nigeria). | Traditional healers comprise herbalists (those who use plant products for medicinal purposes) or diviners (those who claim to gain insight for healing by occultic or ritualistic processes).  Faith healers are those who subscribe to Christian or Islamic faith and rely on prayers and religious rituals, including divination and sacrifices to provide healing.  Primary health care providers (PHCW) consisted of registered nurses, clinical officers, community health officers, or community health extension workers.  Patients are persons with psychotic disorders. | Baseline mean PANSS score was 107.3 (SD17.5) for collaborative shared care (CSC) group and 108.9 (SD18.3) for enhanced care as usual (eCAU) group. 286 (93%) completed the six-month follow-up at which the mean total PANSS score for CSC group was 53.4 (SD19.9), significantly lower than 67.6 (SD 23.3) for eCAU control group. Mean PANSS negative, positive and general psychopathology sub-scale scores were also all much lower for CSC participants. Collaborative shared care led to greater reductions in overall care costs. | The participants were not told their arm of the allocations, but they could have guessed this and the assessments were  based on self-reports. The possibility that the knowledge of the involvement of conventional providers in their care could have influenced the reporting of the outcomes by participants in the CSC arm cannot be excluded.  The judgement about whether a psychotic episode was organic was based on history and physical signs, such as fever and recent head injuries, and not on laboratory investigations. The three-month assessments were not conducted blind for this reason.  Even though the trial was designed to be pragmatic, there were at least two inputs that were not available in routine patient care at the sites: the providers were given incentives to make the visits to the TFH facilities and medications were provided free for the purpose of the trial. |
| 12 | Nyame *et al*. (2021)  Ghana | Focus group discussions (FGDs). | Eight focus group discussions. | All eight FGDs were conducted across the two regions, with each discussion group composed of seven to 12 participants. These were made up of 23 PHC workers, 28 traditional health practitioners [THPs] (14 faith healers, and 14 traditional healers) and 16 service users and their caregivers,  (eight service users and eight caregivers). | Approval of forging partnerships; there were mutual undertones of suspicion.  MM providers were mainly concerned that TM providers may do harm to service users (e.g., through delays in care pathways and human rights abuses).  Service users and their caregivers highlighted the failure of MM to meet their healthcare needs.  Challenges to collaborations, including the lack of options to adequately deal with human rights issues such as chaining and exposure to the vagaries of the weather.  Frequent shortage of psychotropic medication at MM settings. | Service users and their caregivers mainly comprised those who were currently accessing THP services. All  discussions were conducted in the THPs’ setting and this may have affected discussion responses as participants may not have felt comfortable criticising their treatment.  Another limitation of the study was that it focused on service users with psychosis only, although several of the findings may also be generalisable to persons with other forms of mental illness. |
|  |  |  |  |  |  |  |

* Data on the country not included
